# EASIX and CPSS Cytogenetics score-based composite risk model for patients with CMML undergoing allogeneic transplant

**DOI:** 10.1038/s41409-023-02184-0

**Published:** 2024-01-13

**Authors:** Anmol Baranwal, Abhishek Mangaonkar, Mithun V. Shah, Aref Al-Kali, Ayalew Tefferi, William J. Hogan, Mark R. Litzow, Mrinal M. Patnaik, Hassan B. Alkhateeb

**Affiliations:** 1 Division of Hematology, Department of Medicine, Mayo Clinic, Rochester, MN USA; 2 Cancer Centers of Southwest Oklahoma, Lawton, OK USA

**Keywords:** Medical research, Health care

## To the Editor:

Allogeneic stem cell transplant (alloSCT) remains the only potentially curative option for patients with chronic myelomonocytic leukemia (CMML). Studies evaluating alloSCT outcomes in patients with CMML have shown a treatment-related mortality ranging from 12 to 52% [1–6]. The post-alloSCT survival primarily depends on the risk of relapse and non-relapse mortality (NRM). Koenecke et al. showed that the CMML-specific prognostic scoring system (CPSS) cytogenetic risk can predict post-alloSCT relapse [7]. Our group showed that a high Endothelial Activation and Stress Index (EASIX) score was associated with NRM in CMML (HR 3.88, 95% CI 1.53–9.88, *P* = 0.004) [8]. Our goal was to determine a composite risk model using the CPSS cytogenetic risk and EASIX score to predict post-alloSCT overall survival (OS) in patients with CMML.

**Fig. 1:**
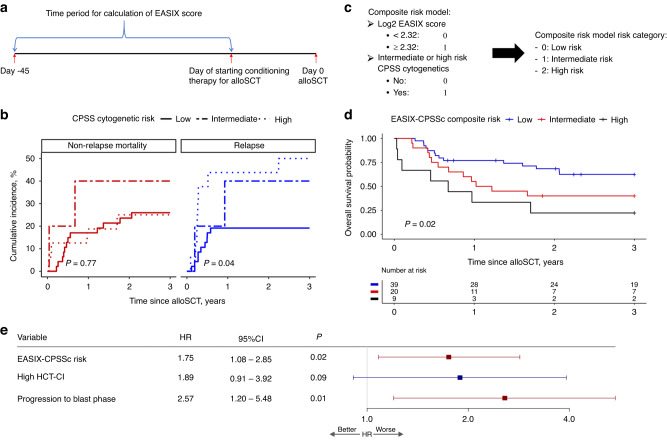
Calculation of EASIX score, composite risk model and survival outcomes in patients with CMML undergoing alloSCT. **a** Time period for calculation of EASIX scores. **b** Relapse incidence in patients with CMML stratified by CPSS cytogenetic risk. **c** EASIX-CPSSc based composite risk model. **d** Survival outcomes stratified by EASIX-CPSSc composite risk model. **e** Multivariate analysis for 3-year survival after allogeneic transplant.

Patients with CMML who underwent alloSCT at Mayo Clinic Rochester between November 1992 and October 2021 were included. EASIX score was calculated using the formula: lactate dehydrogenase (U/L) × Creatinine (mg/dL) / platelet count (10^9^/L) and analyzed on log2-transformed values. The LDH, creatinine and platelet values available on the day of or prior to starting conditioning therapy, within day 45 of alloSCT, were used for calculation of the EASIX score (Fig. 1a). As shown previously by our group, a log2-EASIX score of ≥2.32, which corresponds to a calculated (non-transformed) EASIX score of 5, was considered a high-risk predictor of NRM [8]. CPSS cytogenetic risk category was determined as high risk (trisomy 8, chromosome 7 abnormalities or complex karyotype), low risk (normal karyotype or -Y) or intermediate risk (all others) [7]. An HCT-CI score ≥3 was considered high. Data on patient, disease and transplant characteristics, and post-transplant outcomes were collected retrospectively. Relapse was defined as detection of disease, either morphologic or molecular, after alloSCT. The cumulative incidence of relapse was determined using competing risk analyses, with non-relapse mortality considered as competing risk. Overall survival from transplant was determined using Kaplan–Meier and log-rank method. Median follow-up time was determined using the reverse Kaplan–Meier method. Cox-proportional hazard model was used to determine factors influencing survival post-alloSCT. Variables found significant in univariate analysis at *P* ≤ 0.10 were included in multivariate analysis.

A total of 68 patients (68% males) were evaluated, 51 (75%) of whom had chronic/accelerated phase CMML, while 17 (25%) had progressed to blast phase before alloSCT. Median age at diagnosis was 60 years (IQR 51–64 years). Forty-six (67.6%) patients had CMML-1, while 22 (32.4%) patients had CMML-2, as defined by the 2022 WHO classification [9]. Thirty-two (47.1%) patients met criteria for myelodysplastic CMML, while 36 (52.9%) patients met criteria for myeloproliferative CMML at diagnosis. Thirty-five (51.5%) patients had NGS testing performed. The most common mutations were in *ASXL1* (19/35, 54.3%), *SRSF2* (13/35, 37.1%), and *TET2* (10/35, 28.6%) genes (Supplementary Fig. S1). Of the entire cohort, 18 (26.5%) patients were in complete remission at the time of alloSCT. Thirty (44.1%) patients had an HCT-CI score ≥3.

A total of 17 (25%) patients had a high log2-EASIX score and 51 (75%) had low log2-EASIX score. Forty-seven (69.1%) patients had a low CPSS cytogenetic risk, 5 (7.4%) had intermediate cytogenetic risk, while 16 (23.5%) patients had a high CPSS cytogenetic risk. Competing risk analysis showed that the relapse rate increased with increasing CPSS cytogenetic risk, with a 3-year cumulative incidence of relapse of 19.1% for low risk, 40% for intermediate risk and 50% for patients with high CPSS cytogenetic risk (*P* = 0.04) (Fig. 1b). The competing risk regression analysis showed that relapse risk was significantly higher in patients with an intermediate or high CPSS cytogenetic risk compared to patients with low CPSS cytogenetic risk (HR 3.02, 95% CI 1.25–7.29, *P* = 0.01). Since the high log2-EASIX score is associated with an increased risk of NRM and intermediate/high CPSS cytogenetic risk is associated with an increased risk relapse, we assigned 1 point to the presence of each of these two factors to develop EASIX-CPSS cytogenetic (EASIX-CPSSc) composite risk model. Thereby stratifying patients into three categories: low risk (low-risk cytogenetics + low log2-EASIX score), high risk (intermediate/high-risk cytogenetics + high log2-EASIX score), and intermediate risk (all others) (Fig. 1c).

Median follow-up time after alloSCT was 5.5 years (95% CI 4.99–12.3). Median survival of the entire cohort after alloSCT was 3.2 years (95% CI 1.37–13.6). A total of 39 (57.4%) patients had low composite risk, 20 (29.4%) had intermediate risk and 9 (13.2%) had high composite risk (Supplementary Table 1). Patients with high composite risk had the worst 3-year OS followed by intermediate and low risk patients (3-year OS 22.2% vs. 40% vs. 62.4%, *P* = 0.02, Fig. 1d). Univariate analysis showed that an increasing EASIX-CPSSc composite risk score was associated with worse 3-year survival post-alloSCT (HR 1.88, 95% CI 1.21–2.92, *P* = 0.005). C-statistic for the composite risk model was 0.63.

The EASIX-CPSSc composite risk score, a high HCT-CI score (≥3), and progression to blast phase before alloSCT were significantly associated with worse 3-year OS post-alloSCT (*P* < 0.10, Supplementary Table 2), and were included in the multivariate analysis. Multivariate analysis showed that only EASIX-CPSSc composite risk scoring system (HR 1.75, 95% CI 1.08–2.85, *P* = 0.02, Fig. 1e) and progression to blast phase prior to alloSCT were independent predictors of survival at 3-years post-alloSCT. Since transplant practices changed over time, we also included alloSCT from 2011 onwards as a variable in the multivariate analysis and it did not impact survival (Supplementary Table 3).

Gagelmann et al. have recently proposed a CMML transplant score model to predict post-transplant NRM [10]. The model incorporates ASXL1 or NRAS mutations, bone marrow blast % and HCT-CI score. Patients who had progressed to blast phase or who did not have genetic information available were excluded in the study. A trend toward inferior survival was seen in patient with high HCT-CI score in our study. Mei et al. recently showed that somatic mutations had only a limited impact on post-alloSCT outcomes, with *TP53* mutation being associated with an increased risk of relapse [11]. However, only 3% of patients in the study had a *TP53* mutation [11]. Few other studies have suggested that complete remission before alloSCT may have a positive effect on survival [2, 12]. For instance Symeonidis et al. reported a significantly longer relapse free survival for patients who had alloSCT while in complete remission (median 20.8 vs. 7.6 months, *P* = 0.001) [2]. Our study did not show a significant association of disease status with survival after alloSCT except for blast phase CMML.

Some of the limitations of our study include a small sample size, only a few patients receiving post-transplant cyclophosphamide, and a prolonged accrual time spanning across 30 years. Furthermore, the log2-EASIX score cut-off of 2.32 requires formal validation in larger studies. NGS data were available in only a few patients; therefore, we could not assess the impact of specific somatic gene mutations on post-alloSCT survival; however, studies have shown only a limited impact of somatic gene mutations on post-alloSCT relapse [10, 11].

In summary, our study shows that a composite risk model incorporating pre-conditioning EASIX score and CPSS cytogenetic risk can independently predict survival in patients with CMML undergoing allogeneic transplant. Patients with high composite risk score should be carefully evaluated before proceeding with transplantation. Larger studies with different statistical methodology are needed to confirm these findings.

### Supplementary information

Supplementary material

## Data Availability

The datasets generated during and/or analyzed during the current study are available from the corresponding author upon reasonable request.
