# Supplementary material for: EASIX and CPSS Cytogenetics score-based composite risk model for patients with CMML undergoing allogeneic transplant
Source: Bone Marrow Transplant. 2024 Jan 13;59(4):558–60. doi: 10.1038/s41409-023-02184-0 (PMC10994838; doi:10.1038/s41409-023-02184-0)
Supplement: Supplementary file 1 — Supplementary material [file 41409_2023_2184_MOESM1_ESM.docx]

**Supplemental Table 1.** Characteristics and outcomes of patients with CMML stratified by EASIX-CPSSc composite risk model.

|  | **Low (N=39)** | **Intermediate (N=20)** | **High (N=9)** | ***P*** |
| --- | --- | --- | --- | --- |
| **Age at alloSCT** |  |  |  |  |
| Median [Min, Max] | 60.5 [35.2, 73.5] | 64.9 [18.3, 73.0] | 58.4 [32.1, 63.9] |  |
| **Gender** |  |  |  |  |
| Female | 16 (41.0%) | 4 (20.0%) | 2 (22.2%) | 0.21 |
| Male | 23 (59.0%) | 16 (80.0%) | 7 (77.8%) |  |
| **CMML type** |  |  |  |  |
| CMML-1 | 28 (71.8%) | 14 (70.0%) | 4 (44.4%) | 0.28 |
| CMML-2 | 11 (28.2%) | 6 (30.0%) | 5 (55.6%) |  |
| **CMML type (by WBC)** |  |  |  |  |
| Myelodysplastic | 18 (46.2%) | 9 (45.0%) | 5 (55.6%) | 0.86 |
| Myeloproliferative | 21 (53.8%) | 11 (55.0%) | 4 (44.4%) |  |
| **Splenomegaly** |  |  |  |  |
| No | 32 (82.1%) | 17 (85.0%) | 9 (100%) | 0.39 |
| Yes | 7 (17.9%) | 3 (15.0%) | 0 (0%) |  |
| **CR at alloSCT** |  |  |  |  |
| No | 30 (76.9%) | 15 (75.0%) | 4 (44.4%) | 0.29 |
| Yes | 9 (23.1%) | 5 (25.0%) | 4 (44.4%) |  |
| Missing | 0 (0%) | 0 (0%) | 1 (11.1%) |  |
| **Progression to blast phase** |  |  |  |  |
| No | 31 (79.5%) | 16 (80.0%) | 4 (44.4%) | 0.08 |
| Yes | 8 (20.5%) | 4 (20.0%) | 5 (55.6%) |  |
| **HCT-CI ≥ 3** |  |  |  |  |
| No | 23 (59.0%) | 9 (45.0%) | 4 (44.4%) | 0.68 |
| Yes | 16 (41.0%) | 10 (50.0%) | 4 (44.4%) |  |
| Missing | 0 (0%) | 1 (5.0%) | 1 (11.1%) |  |
| **Graft source** |  |  |  |  |
| BM | 6 (15.4%) | 2 (10.0%) | 2 (22.2%) | 0.11 |
| DUCB | 0 (0%) | 0 (0%) | 1 (11.1%) |  |
| PBSC | 33 (84.6%) | 18 (90.0%) | 6 (66.7%) |  |
| **Donor source** |  |  |  |  |
| MRD | 13 (33.3%) | 8 (40.0%) | 4 (44.4%) | 0.79 |
| MUD | 20 (51.3%) | 11 (55.0%) | 4 (44.4%) |  |
| Others | 6 (15.4%) | 1 (5.0%) | 1 (11.1%) |  |
| **Reduced-intensity conditioning** |  |  |  |  |
| No | 14 (35.9%) | 6 (30.0%) | 5 (55.6%) | 0.41 |
| Yes | 25 (64.1%) | 14 (70.0%) | 4 (44.4%) |  |
| **Major ABO mismatch** |  |  |  |  |
| No | 32 (82.1%) | 16 (80.0%) | 6 (66.7%) | 0.85 |
| Yes | 7 (17.9%) | 3 (15.0%) | 2 (22.2%) |  |
| Missing | 0 (0%) | 1 (5.0%) | 1 (11.1%) |  |
| **CMV (both recipient/donor negative)** |  |  |  |  |
| No | 14 (35.9%) | 6 (30.0%) | 0 (0%) | 0.11 |
| Yes | 23 (59.0%) | 13 (65.0%) | 8 (88.9%) |  |
| Missing | 2 (5.1%) | 1 (5.0%) | 1 (11.1%) |  |
| **GVHD prophylaxis** |  |  |  |  |
| Tacrolimus + Methotrexate | 20 (51.3%) | 9 (45%) | 2 (22.2%) | 0.16 |
| Tacrolimus + MMF | 1 (2.6%) | 0 (0%) | 0 (0%) |  |
| Cyclosporine + Methotrexate | 13 (33.3%) | 9 (45%) | 4 (44.4%) |  |
| Cyclosporine ± MMF | 3 (7.8%) | 0 (0%) | 3 (33.3%) |  |
| PT-Cy based | 2 (5.2%) | 2 (10%) | 0 (0%) |  |
| **Acute GVHD grade 2-4** |  |  |  |  |
| No | 24 (61.5%) | 12 (60.0%) | 5 (55.6%) | 0.99 |
| yes | 15 (38.5%) | 8 (40.0%) | 3 (33.3%) |  |
| Missing | 0 (0%) | 0 (0%) | 1 (11.1%) |  |
| **Acute GVHD grade 3-4** |  |  |  |  |
| No | 29 (74.4%) | 17 (85.0%) | 5 (55.6%) | 0.42 |
| Yes | 10 (25.6%) | 3 (15.0%) | 3 (33.3%) |  |
| Missing | 0 (0%) | 0 (0%) | 1 (11.1%) |  |
| **Chronic GVHD, mod-severe** |  |  |  |  |
| No | 10 (25.6%) | 3 (15%) | 1 (11.1%) | 0.87 |
| Yes | 19 (48.7%) | 8 (40%) | 3 (33.3%) |  |
| Missing | 10 (25.6%) | 9 (45.0%) | 5 (55.6%) |  |
| **Abbreviations:** alloSCT: allogeneic stem cell transplant, BM: bone marrow, CMML: chronic myelomonocytic leukemia, CMV: cytomegalovirus, CR: complete remission, DUCB: double umbilical cord blood, GVHD: graft versus host disease, HCT-CI: Hematopoietic Cell Transplantation-specific Comorbidity Index, PBSC: peripheral blood stem cells, MMF: mycophenolate mofetil, MRD: matched related donor, MUD: matched unrelated donor, PT-Cy: post-transplant cyclophosphamide. | | | | |

**Supplemental Table 2.** Univariate analysis for 3-year survival after transplant.

| **Variable** | **HR** | **95% CI** | ***P*** |
| --- | --- | --- | --- |
| EASIX-CPSSc composite risk model | 1.88 | 1.21-2.92 | **0.005** |
| Male gender | 1.2 | 0.57-2.51 | 0.64 |
| Age at alloSCT > 60 years | 1.07 | 0.54-2.13 | 0.84 |
| alloSCT from year 2011 onwards | 0.59 | 0.30-1.18 | 0.14 |
| Hemoglobin < 10 g/dL at diagnosis | 1.11 | 0.54-2.29 | 0.77 |
| Platelets < 100 x 10^9^ cells/L at diagnosis | 1 | 0.50-2.09 | 0.99 |
| AMC > 10 x 10^9^ cells/L at diagnosis | 0.93 | 0.36-2.43 | 0.88 |
| Circulating IMC | 1.82 | 0.75-4.45 | 0.19 |
| Myeloproliferative subtype | 0.66 | 0.33-1.30 | 0.23 |
| CMML-2 (vs CMML-1) | 0.88 | 0.42-1.85 | 0.73 |
| High HCTCI score (HCT-CI score ≥ 3) | 1.96 | 0.97-3.95 | **0.06** |
| PBSCT graft | 0.96 | 0.37-2.50 | 0.94 |
| RIC | 0.95 | 0.47-1.94 | 0.9 |
| Progression to blast phase before alloSCT | 2.63 | 1.30-5.33 | **0.007** |
| CR at alloSCT | 0.82 | 0.37-1.82 | 0.62 |
| Prior treatment before alloSCT | 1.33 | 0.47-3.80 | 0.59 |
| Matched unrelated (vs matched-related donor) | 1.2 | 0.58-2.48 | 0.63 |
| Busulfan-based conditioning | 1.16 | 0.54-2.50 | 0.7 |
| Both donor/recipient CMV negative | 0.99 | 0.45-2.17 | 0.97 |
| Major ABO mismatch | 1.65 | 0.74-3.71 | 0.22 |
| **Abbreviations:** alloSCT: allogeneic stem cell transplant, AMC: absolute monocyte count, IMC: immature myeloid cells, CMML: chronic myelomonocytic leukemia, CMV: cytomegalovirus, CR: complete response, HCT-CI: Hematopoietic Cell Transplantation-specific Comorbidity Index, PBSCT: peripheral blood stem cell transplant, RIC: reduced-intensity conditioning | | | |

**Supplemental Table 3.** Multivariate analysis for 3-year survival after transplant, including alloSCT from 2011 onwards, as a variable.

| **Variable** | **HR** | **95% CI** | ***P*** |
| --- | --- | --- | --- |
| EASIX-CPSSc composite risk model | 1.73 | 1.06-2.83 | **0.03** |
| Progression to blast phase before alloSCT | 2.54 | 1.18-5.44 | **0.02** |
| High HCTCI score (HCT-CI score ≥ 3) | 1.87 | 0.90-3.90 | 0.10 |
| alloSCT from year 2011 onwards | 0.91 | 0.44-1.90 | 0.80 |

**
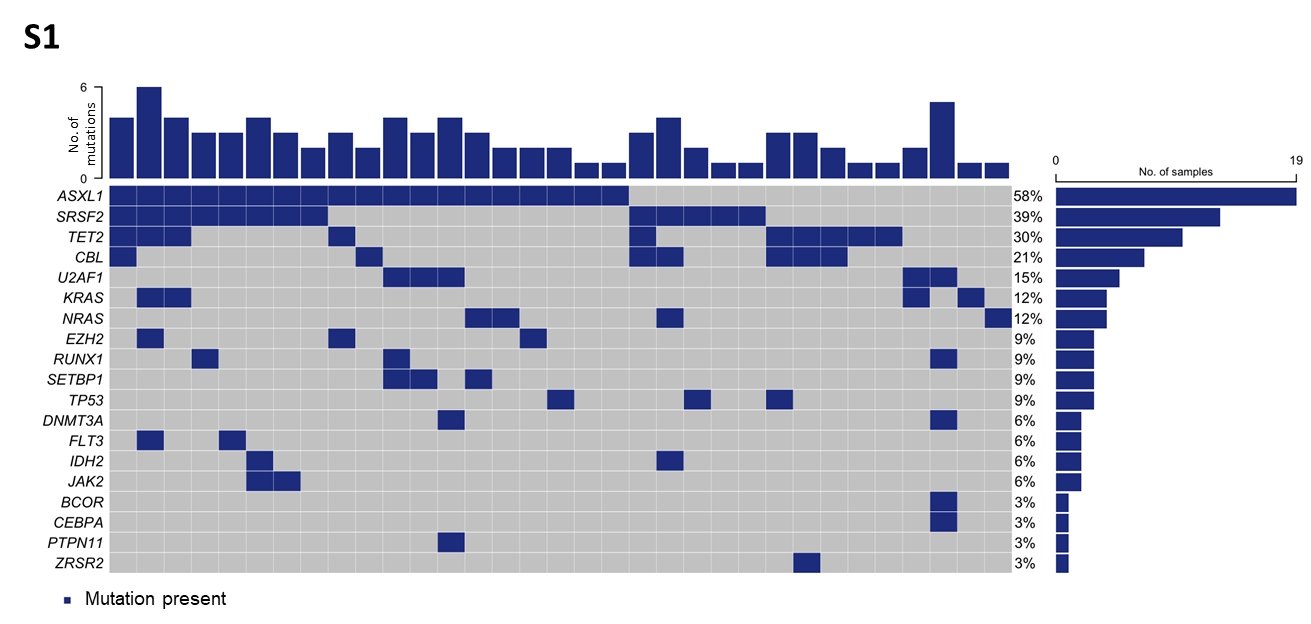
**

**Supplementary Figure S1.** Mutational correlation among patients with CMML who had NGS results available.
